# Crystal structures of isomeric 3,5-di­chloro-*N*-(2,3-di­methyl­phen­yl)benzene­sulfonamide, 3,5-di­chloro-*N*-(2,6-di­methyl­phen­yl)benzene­sulfonamide and 3,5-di­chloro-*N*-(3,5-di­methyl­phen­yl)benzene­sulfonamide

**DOI:** 10.1107/S2056989017005230

**Published:** 2017-04-11

**Authors:** K. Shakuntala, S. Naveen, N. K. Lokanath, P. A. Suchetan

**Affiliations:** a Department of Chemistry, Sri Bhuvanendra College, Karkala 574 104, India; b Institution of Excellence, University of Mysore, Manasagangotri, Mysuru 570 006, India; c Department of Studies in Physics, University of Mysore, Manasagangotri, Mysuru 570 006, India; d Dept. of Chemistry, University College of Science, Tumkur University, Tumkur, 572103, India

**Keywords:** crystal structure, sulfonamides, N—H⋯O hydrogen bonds, C—H⋯O inter­actions, C—H⋯π inter­actions, π–π inter­actions

## Abstract

The crystal structures of three isomeric compounds of formula C_14_H_13_Cl_2_NO_2_S, namely 3,5-di­chloro-*N*-(2,3-di­methyl­phen­yl)-benzene­sulfonamide (I), 3,5-di­chloro-*N*-(2,6-di­methyl­phen­yl)benzene­sulfonamide (II) and 3,5-di­chloro-*N*-(3,5-di­methyl­phen­yl)benzene­sulfonamide (III) are described. The mol­ecules of all the three compounds are U-shaped with the two aromatic rings inclined at 41.3 (6)° in (I), 42.1 (2)° in (II) and 54.4 (3)° in (III). The mol­ecular conformation of (II) is stabilized by intra­molecular C—H⋯O hydrogen bonds and C—H⋯π inter­actions. The crystal structure of (I) features N—H⋯O hydrogen-bonded *R*_2_^2^(8) loops inter­connected *via C*(7) chains of C—H⋯O inter­actions, forming a three-dimensional architecture. The structure also features π–π inter­actions [*Cg*⋯*Cg* = 3.6970 (14) Å]. In (II), N—H⋯O hydrogen-bonded *R*_2_^2^(8) loops are inter­connected *via* π–π inter­actions [inter­centroid distance = 3.606 (3) Å] to form a one-dimensional architecture running parallel to the *a* axis. In (III), adjacent *C*(4) chains of N—H⋯O hydrogen-bonded mol­ecules running parallel to [010] are connected *via* C—H⋯π inter­actions, forming sheets parallel to the *ab* plane. Neighbouring sheets are linked *via* offset π–π inter­actions [inter­centroid distance = 3.8303 (16) Å] to form a three-dimensional architecture.

## Abstract 2

In the isomeric title compounds, N—H⋯O and C—H⋯O hydrogen bond, and C—H⋯π and π–π inter­actions build different supra­molecular architectures.

## Chemical context   

Sulfonamide drugs were the first chemotherapeutic agents to be used for curing and preventing bacterial infection in human beings (Shiva Prasad *et al.*, 2011 ▸). They play a vital role as key constituents in a number of biologically active mol­ecules and are known to exhibit a wide variety of biological activities, such as anti­bacterial (Subhakara Reddy *et al.*, 2012 ▸; Himel *et al.*, 1971 ▸), anti­fungal (Hanafy *et al.*, 2007 ▸), anti-inflammatory (Küçükgüzel *et al.*, 2013 ▸), anti­tumor (Ghorab *et al.*, 2011 ▸), anti­cancer (Al-Said *et al.*, 2011 ▸), anti-HIV (Sahu *et al.*, 2007 ▸) and anti­tubercular activities (Vora & Mehta, 2012 ▸). In recent years, extensive research studies have been carried out on the synthesis and evaluation of the pharmacological properties of mol­ecules containing the sulfonamide moiety, which have been reported to be important pharmacophores (Mohan *et al.*, 2013 ▸).

With these considerations in mind and based on our structural study of 3,5-di­chloro-*N*-(substitutedphen­yl)benzene­sulfonamides (Shakuntala, Naveen *et al.*, 2017 ▸; Shakuntala, Lokanath *et al.*, 2017 ▸), we report herein the crystal structures of three isomers, *viz.* 3,5-di­chloro-*N*-(2,3-di­methyl­phen­yl)-benzene­sulfonamide (I), 3,5-di­chloro-*N*-(2,6-di­methyl­phen­yl)benzene­sulfonamide (II) and 3,5-di­chloro-*N*-(3,5-di­methyl­phen­yl)benzene­sulfonamide (III).
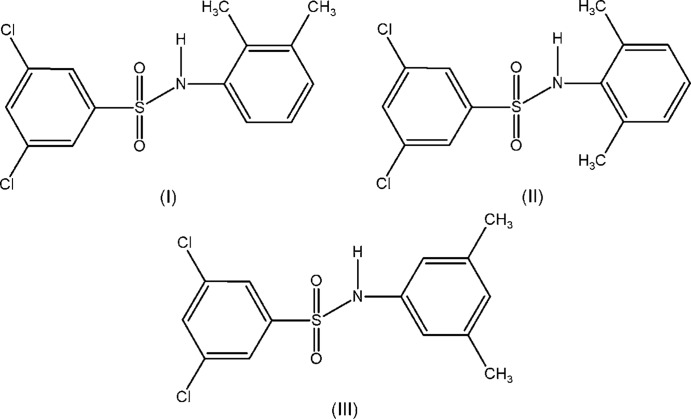


## Structural commentary   

**Figure 1:**
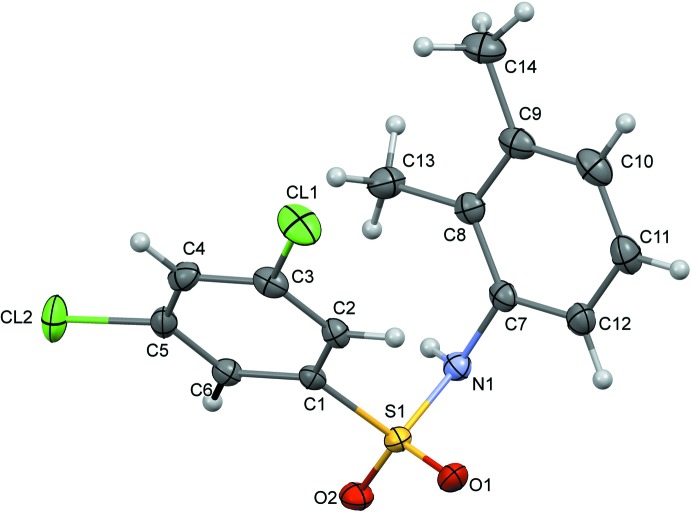
The mol­ecular structure of (I) with displacement ellipsoids drawn at the 50% probability level.

The mol­ecule of (I) (Fig. 1 ▸) is U-shaped, with the sulfonyl­benzene ring and the aniline ring inclined by 41.3 (6)°. The N—C bond in the C–SO_2_–NH–C segment has a *gauche* torsion with respect to the S=O bonds, and the mol­ecule is twisted at the S—N bond, with a C1—S1—N1—C7 torsion angle of 60.9 (2)°.

**Figure 2:**
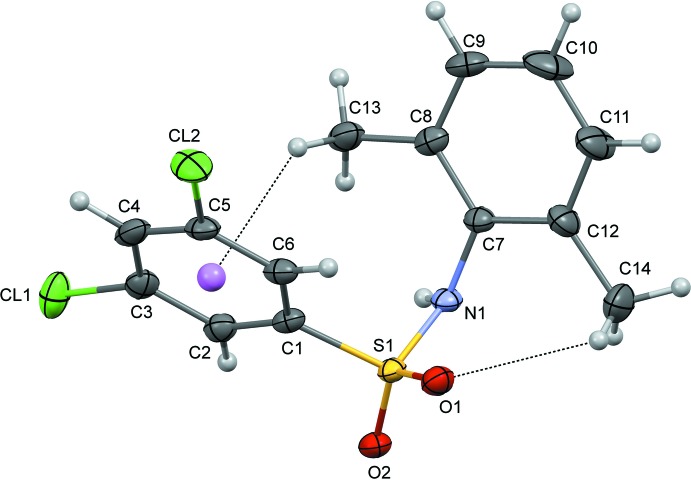
The mol­ecular structure of (II) with displacement ellipsoids drawn at the 50% probability level. Intra­molecular C—H⋯O and C—H⋯π hydrogen inter­actions are shown as dotted lines.

In the U-shaped mol­ecules of (II) (Fig. 2 ▸), the dihedral angle between the sulfonyl­benzene ring and the aniline ring is 42.1 (2)°. The mol­ecule is twisted at the S—N bond, with a C1—S1—N1—C7 torsion angle of 69.8 (3)°. The mol­ecular conformation of (II) is stabilized by an intra­molecular C—H⋯O hydrogen bond and a C—H⋯π inter­action (Table 2 ▸). The N—C bond in the C–SO_2_–NH–C segment has a *gauche* torsion with respect to the S=O bonds.

**Figure 3:**
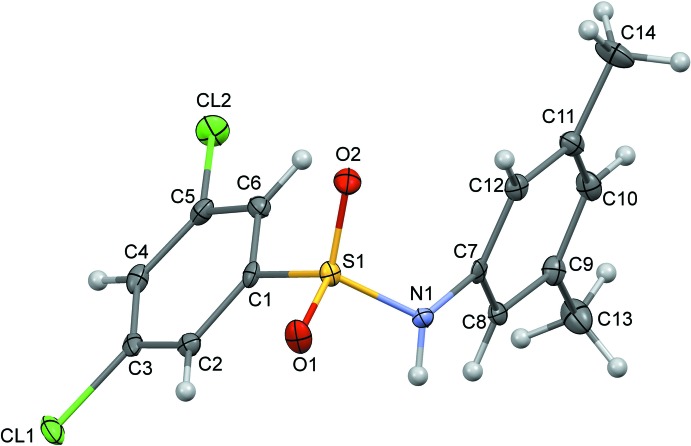
The mol­ecular structure of (III) with displacement ellipsoids drawn at the 50% probability level.

The mol­ecule of (III) (Fig. 3 ▸) is also U-shaped, with the sulfonyl­benzene ring tilted at an angle of 54.4 (3)° with respect to the aniline ring. The N—C bond in the C–SO_2_–NH–C segment has a *gauche* torsion with respect to the S=O bonds, and the mol­ecule is twisted at the S—N bond, with a C1—S1—N1—C7 torsion angle of 71.3 (2)°.

## Supra­molecular features   

**Figure 4:**
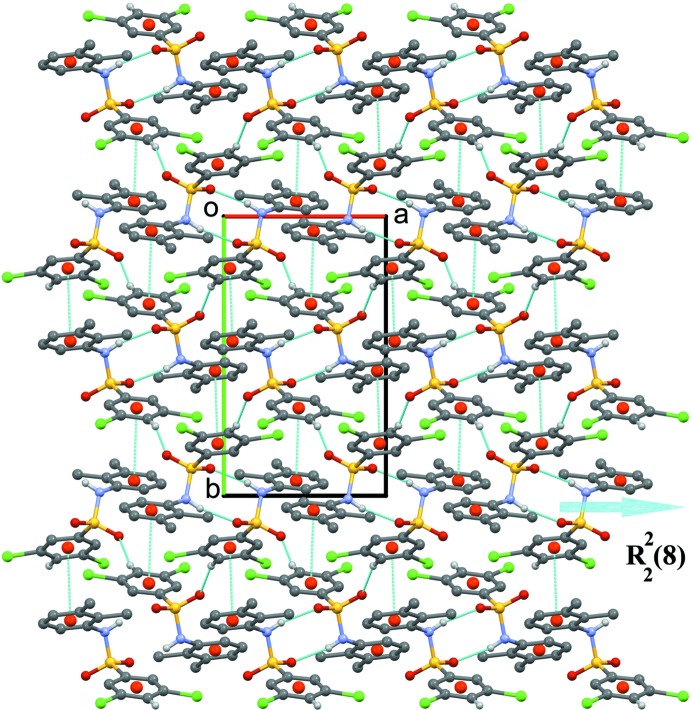
The three-dimensional supra­molecular architecture of (I) viewed along the *c* axis. The N—H⋯O and C—H⋯O hydrogen bonds and π–π inter­actions are shown as thin blue dotted lines. H atoms not involved in hydrogen bonding are omitted for clarity.

The crystal structure of (I) features inversion-related dimers linked by N1—H1⋯O2^i^ hydrogen bonds forming 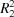
(8) loops (Fig. 4 ▸, Table 1 ▸). The 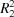
(8) loops are inter­connected *via C*(7) chains of C4—H4⋯O1^ii^ inter­molecular inter­actions, forming a three-dimensional supra­molecular architecture. The structure also features π–π inter­actions involving the benzene­sulfonyl ring and the aniline ring as illustrated in Fig. 4 ▸ [*Cg*1⋯*Cg*2^iii^ = 3.6970 (14) Å; *Cg*1 and *Cg*2 are the centroids of the C1–C6 and C7–C12 rings, respectively; symmetry code: (iii) 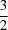 − *x*, −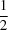 + *y*, 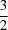 − *z*].

**Figure 5:**
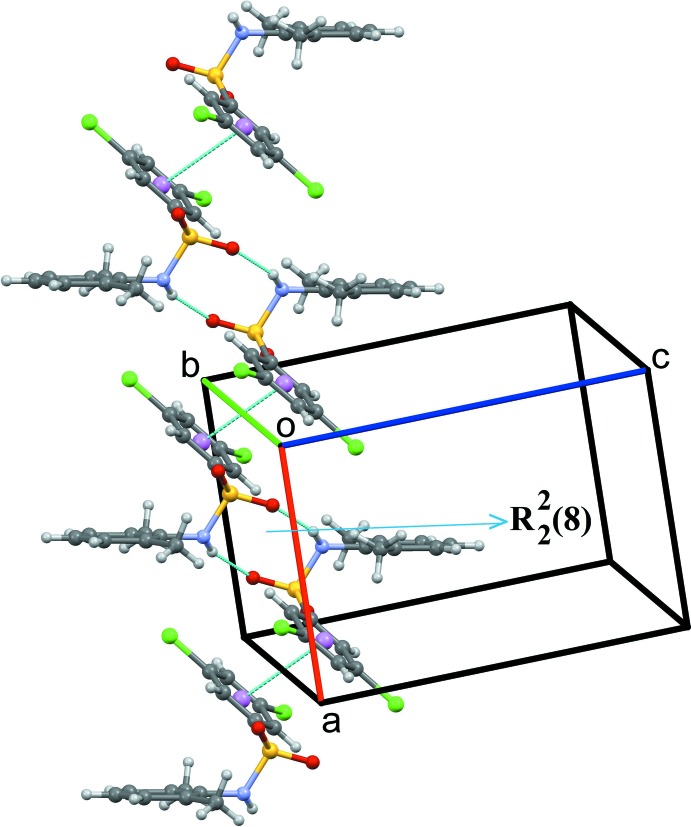
Partial crystal packing of (II) showing the formation of a one-dimensional architecture through N—H⋯O hydrogen bonds and π–π inter­actions (thin blue dotted lines).

In (II), N1—H1⋯O2^i^ hydrogen-bonded 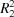
(8) loops (Fig. 5 ▸, Table 2 ▸) are connected *via* π–π inter­actions involving inversion-related benzene­sulfonyl rings, forming a one-dimensional architecture running parallel to the *a* axis, as shown in Fig. 5 ▸ [*Cg*1⋯*Cg*1^ii^ = 3.606 (3) Å; *Cg*1 is the centroid of the C1–C6 ring; symmetry code: (ii) 2 − *x*, 1 − *y*, −*z*].

**Figure 6:**
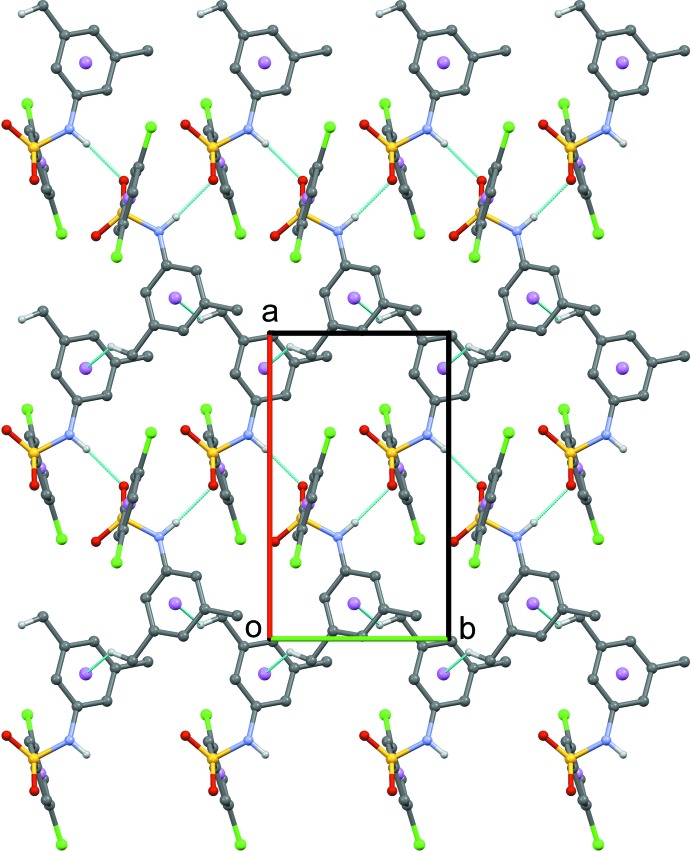
Partial crystal packing of (III) viewed down the *c* axis displaying two-dimensional sheets. Thin blue dotted lines denote N—H⋯O hydrogen bonds and C—H⋯π inter­actions. H atoms not involved in hydrogen bonding are omitted for clarity.

**Figure 7:**
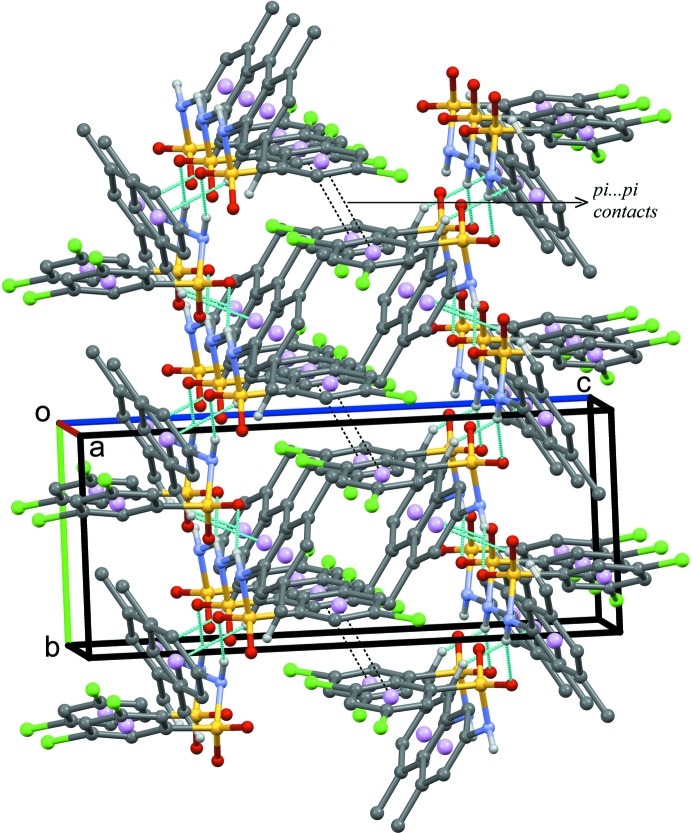
Crystal packing of (III) viewed approximately along the *a* axis, showing the π–π inter­actions (black dotted lines) between adjacent sheets. For clarity, only H atoms involved in N—H⋯O hydrogen bonds and C—H⋯π inter­actions (thin blue dotted lines) are included.

In the crystal structure of (III), the mol­ecules are inter­linked *via* N1—H1⋯O1^i^ hydrogen bonds (Fig. 6 ▸, Table 3 ▸) to form *C*(4) chains running parallel to [010]. Adjacent chains are connected by C14—H14*B*⋯π inter­actions involving the aniline ring, forming two-dimensional sheets parallel to the *ab* plane. Neighbouring sheets are further linked *via* offset π–π inter­actions involving inversion-related benzene­sulfonyl rings, forming a three dimensional architecture as as illustrated in Fig. 7 ▸ [*Cg*1⋯*Cg*1^i^ = 3.8303 (16) Å, inter­planar distance = 3.3874 (11) Å, slippage 1.788 (3) Å; *Cg*1 is the centroid of the C1–C6 ring; symmetry code: (iii) 1 − *x*, −*y*, −*z*].

## Database survey   

Two 3,5-di­chloro-*N*-(substitutedphen­yl)-benzene­sulfon­amides, namely 3,5-di­chloro-*N*-(4-methyl­phen­yl)benzene­sulfonamide [Shakuntala, Naveen *et al.*, 2017 ▸, (IV)] and 3,5-di­chloro-*N*-(2,4-di­chloro­phen­yl)benzene­sulfonamide [Shak­untala, Lokanath *et al.*, 2017 ▸, (V)], have been reported previously. The mol­ecules of both (IV) and (V) are U-shaped with the central C–S–N–C segment having a torsion angle of 67.2 (4)° in (IV) and 58.7 (3)° in (V). The dihedral angle between the benzene rings is 57.0 (2)° in (IV) and 40.23 (2)° in (V). The crystal structure of (IV) displays a three-dimensional supra­molecular structure constructed *via* N—H⋯O and C—H⋯O hydrogen bonds and C—H⋯π inter­actions, whereas in (V) the three-dimensional supra­molecular architecture is built through N—H⋯O and C—H⋯O hydrogen bonds, Cl⋯Cl contacts and π–π inter­actions.

## Synthesis and crystallization   

The title compounds were prepared according to a literature method (Rodrigues *et al.*, 2015 ▸). The purities of all the compounds were checked by determining their melting points. Colourless prismatic single crystals suitable for X-ray diffraction studies were obtained by slow evaporation of ethano­lic solutions of the compounds at room temperature.

## Refinement details   

Crystal data, data collection and structure refinement details are summarized in Table 4 ▸. The amino H atoms were located in difference-Fourier maps and refined isotropically with the N—H bond length restrained to be 0.88 (2) Å. All other H atoms were positioned geometrically and refined as riding with C—H = 0.95–0.98 Å and *U*_iso_(H) = 1.2 or 1.5*U*_eq_(C). A rotating model was applied to the methyl groups. To improve considerably the values of *R*1, *wR*2, and *S* (goodness-of-fit), a low-angle reflection partially obscured by the beam-stop (100) was omitted from the final refinement of (III).

## Supplementary Material

Crystal structure: contains datablock(s) I, II, III, shelx. DOI: 10.1107/S2056989017005230/rz5211sup1.cif

Structure factors: contains datablock(s) I. DOI: 10.1107/S2056989017005230/rz5211Isup2.hkl

Structure factors: contains datablock(s) II. DOI: 10.1107/S2056989017005230/rz5211IIsup3.hkl

Structure factors: contains datablock(s) III. DOI: 10.1107/S2056989017005230/rz5211IIIsup4.hkl

Supporting information file. DOI: 10.1107/S2056989017005230/rz5211Isup5.cml

Supporting information file. DOI: 10.1107/S2056989017005230/rz5211IIsup6.cml

Supporting information file. DOI: 10.1107/S2056989017005230/rz5211IIIsup7.cml

CCDC references: 1542706, 1542704, 1542705

Additional supporting information:  crystallographic information; 3D view; checkCIF report

## Figures and Tables

**Table 1:** Hydrogen-bond geometry (Å, °) for (I)

*D*—H⋯*A*	*D*—H	H⋯*A*	*D*⋯*A*	*D*—H⋯*A*
N1—H1⋯O2^i^	0.86	2.14	2.9590	159
C4—H4⋯O1^ii^	0.95	2.41	3.332 (3)	164

Symmetry codes: (i) 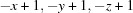; (ii) 
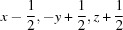
.

**Table 2:** Hydrogen-bond geometry (Å, °) for (II) *Cg*1 is the centroid of the C1–C6 ring.

*D*—H⋯*A*	*D*—H	H⋯*A*	*D*⋯*A*	*D*—H⋯*A*
C14—H14*C*⋯O1	0.98	2.53	3.139 (8)	120
N1—H1⋯O2^i^	0.85 (4)	2.12 (4)	2.937 (5)	160 (4)
C13—H13*A*⋯*Cg*1	0.98	2.67	3.493 (5)	142

Symmetry code: (i) 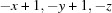.

**Table 3:** Hydrogen-bond geometry (Å, °) for (III) *Cg*2 is the centroid of the aniline ring C7–C12

*D*—H⋯*A*	*D*—H	H⋯*A*	*D*⋯*A*	*D*—H⋯*A*
N1—H1⋯O1^i^	0.87	2.13	2.9848	167
C14—H14*B*⋯*Cg*2^ii^	0.98	2.86	3.5135	124

Symmetry codes: (i) 
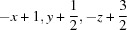
; (ii) 
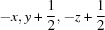
.

**Table 4:** Experimental details

	(I)	(II)	(III)
Crystal data			
Chemical formula	C_14_H_13_Cl_2_NO_2_S	C_14_H_13_Cl_2_NO_2_S	C_14_H_13_Cl_2_NO_2_S
*M*_r_	330.21	330.21	330.21
Crystal system, space group	Monoclinic, *P*2_1_/*n*	Triclinic, *P* 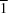	Monoclinic, *P*2_1_/*c*
Temperature (K)	100	100	100
*a*, *b*, *c* (Å)	8.2223 (3), 14.1546 (5), 12.7933 (4)	8.4817 (15), 8.6149 (15), 12.167 (2)	12.2268 (6), 7.0399 (3), 17.3130 (8)
α, β, γ (°)	90, 91.188 (1), 90	109.875 (5), 91.900 (5), 114.190 (5)	90, 100.409 (1), 90
*V* (Å^3^)	1488.61 (9)	747.1 (2)	1465.70 (12)
*Z*	4	2	4
Radiation type	Cu *K*α	Cu *K*α	Cu *K*α
μ (mm^−1^)	5.24	5.22	5.32
Crystal size (mm)	0.28 × 0.25 × 0.22	0.29 × 0.26 × 0.22	0.27 × 0.24 × 0.21

Data collection			
Diffractometer	Bruker APEXII CCD area detector	Bruker APEXII CCD area detector	Bruker APEXII CCD area detector
Absorption correction	Multi-scan (*SADABS*; Bruker, 2009 ▸)	Multi-scan (*SADABS*; Bruker, 2009 ▸)	Multi-scan (*SADABS*; Bruker,2009 ▸)
*T*_min_, *T*_max_	0.288, 0.316	0.275, 0.317	0.297, 0.327
No. of measured, independent and observed [*I* > 2σ(*I*)] reflections	10308, 2440, 2347	6977, 2400, 1960	11468, 2412, 2374
*R*_int_	0.053	0.124	0.056
(sin θ/λ)_max_ (Å^−1^)	0.584	0.581	0.585

Refinement			
*R*[*F* ^2^ > 2σ(*F* ^2^)], *wR*(*F* ^2^), *S*	0.057, 0.162, 1.07	0.074, 0.233, 1.02	0.058, 0.152, 0.99
No. of reflections	2440	2400	2412
No. of parameters	187	187	187
No. of restraints	1	1	1
H-atom treatment	H atoms treated by a mixture of independent and constrained refinement	H atoms treated by a mixture of independent and constrained refinement	H atoms treated by a mixture of independent and constrained refinement
Δρ_max_, Δρ_min_ (e Å^−3^)	0.64, −0.63	0.99, −0.60	0.82, −0.88

Computer programs: *APEX2*, *SAINT-Plus* and *XPREP* (Bruker, 2009 ▸), *SHELXT* 2016/4 (Sheldrick, 2015*a* ▸), *SHELXL2016/4* (Sheldrick, 2015*b* ▸) and *Mercury* (Macrae *et al.*, 2008 ▸).

## supplementary crystallographic information

### (I) 3,5-Dichloro-N-(2,3-dimethylphenyl)benzenesulfonamide Crystal data

**Table:**

C_14_H_13_Cl_2_NO_2_S	Prism
*M**_r_* = 330.21	*D*_x_ = 1.473 Mg m^−^^3^
Monoclinic, *P*2_1_/*n*	Melting point: 431 K
Hall symbol: -P 2yn	Cu *K*α radiation, λ = 1.54178 Å
*a* = 8.2223 (3) Å	Cell parameters from 144 reflections
*b* = 14.1546 (5) Å	θ = 6.2–64.2°
*c* = 12.7933 (4) Å	µ = 5.24 mm^−^^1^
β = 91.188 (1)°	*T* = 100 K
*V* = 1488.61 (9) Å^3^	Prism, colourless
*Z* = 4	0.28 × 0.25 × 0.22 mm
*F*(000) = 680	

### (I) 3,5-Dichloro-N-(2,3-dimethylphenyl)benzenesulfonamide Data collection

**Table:**

Bruker APEXII CCD area detector diffractometer	2440 independent reflections
Radiation source: fine-focus sealed tube	2347 reflections with *I* > 2σ(*I*)
Graphite monochromator	*R*_int_ = 0.053
phi and φ scans	θ_max_ = 64.2°, θ_min_ = 6.2°
Absorption correction: multi-scan (SADABS; Bruker, 2009)	*h* = −9→9
*T*_min_ = 0.288, *T*_max_ = 0.316	*k* = −16→15
10308 measured reflections	*l* = −14→12

### (I) 3,5-Dichloro-N-(2,3-dimethylphenyl)benzenesulfonamide Refinement

**Table:**

Refinement on *F*^2^	1 restraint
Least-squares matrix: full	Hydrogen site location: mixed
*R*[*F*^2^ > 2σ(*F*^2^)] = 0.057	H atoms treated by a mixture of independent and constrained refinement
*wR*(*F*^2^) = 0.162	*w* = 1/[σ^2^(*F*_o_^2^) + (0.1235*P*)^2^ + 0.7281*P*] where *P* = (*F*_o_^2^ + 2*F*_c_^2^)/3
*S* = 1.07	(Δ/σ)_max_ < 0.001
2440 reflections	Δρ_max_ = 0.64 e Å^−^^3^
187 parameters	Δρ_min_ = −0.63 e Å^−^^3^

### (I) 3,5-Dichloro-N-(2,3-dimethylphenyl)benzenesulfonamide Special details

**Table:**

Geometry. All esds (except the esd in the dihedral angle between two l.s. planes) are estimated using the full covariance matrix. The cell esds are taken into account individually in the estimation of esds in distances, angles and torsion angles; correlations between esds in cell parameters are only used when they are defined by crystal symmetry. An approximate (isotropic) treatment of cell esds is used for estimating esds involving l.s. planes.

### (I) 3,5-Dichloro-N-(2,3-dimethylphenyl)benzenesulfonamide Fractional atomic coordinates and isotropic or equivalent isotropic displacement parameters (Å^2^)

**Table:**

	*x*	*y*	*z*	*U*_iso_*/*U*_eq_	
C1	0.6064 (3)	0.35091 (16)	0.66816 (18)	0.0170 (5)	
C2	0.7069 (3)	0.32517 (16)	0.75161 (18)	0.0189 (5)	
H2	0.821571	0.332433	0.748662	0.023*	
C3	0.6346 (3)	0.28862 (18)	0.83908 (19)	0.0222 (6)	
C4	0.4689 (3)	0.27523 (17)	0.84477 (19)	0.0230 (6)	
H4	0.421903	0.248689	0.905281	0.028*	
C5	0.3733 (3)	0.30179 (17)	0.7592 (2)	0.0214 (6)	
C6	0.4380 (3)	0.34137 (16)	0.67041 (19)	0.0191 (5)	
H6	0.370447	0.361192	0.613395	0.023*	
C7	0.8409 (3)	0.53406 (16)	0.67541 (19)	0.0200 (5)	
C8	0.7774 (3)	0.56423 (17)	0.7703 (2)	0.0214 (6)	
C9	0.8878 (3)	0.58319 (17)	0.8534 (2)	0.0255 (6)	
C10	1.0528 (3)	0.57152 (19)	0.8392 (2)	0.0299 (6)	
H10	1.126374	0.583905	0.895815	0.036*	
C11	1.1130 (3)	0.5422 (2)	0.7444 (2)	0.0294 (6)	
H11	1.226895	0.535041	0.736077	0.035*	
C12	1.0071 (3)	0.52333 (18)	0.6620 (2)	0.0249 (6)	
H12	1.047530	0.503204	0.596579	0.030*	
C13	0.5980 (3)	0.5761 (2)	0.7852 (2)	0.0291 (6)	
H13A	0.542286	0.578976	0.716866	0.044*	
H13B	0.578180	0.634742	0.823689	0.044*	
H13C	0.556474	0.522395	0.824971	0.044*	
C14	0.8264 (4)	0.6143 (2)	0.9586 (2)	0.0349 (7)	
H14A	0.918902	0.623555	1.007098	0.052*	
H14B	0.754217	0.565655	0.986298	0.052*	
H14C	0.766435	0.673737	0.950595	0.052*	
N1	0.7344 (3)	0.51231 (14)	0.58726 (16)	0.0194 (5)	
O1	0.8517 (2)	0.35517 (12)	0.54650 (13)	0.0237 (4)	
O2	0.5832 (2)	0.40294 (13)	0.47232 (14)	0.0241 (4)	
S1	0.69924 (7)	0.40208 (4)	0.55760 (4)	0.0177 (3)	
CL1	0.75874 (9)	0.25743 (5)	0.94545 (5)	0.0355 (3)	
CL2	0.16538 (7)	0.28217 (5)	0.76316 (6)	0.0356 (3)	
H1	0.647 (3)	0.5457 (18)	0.585 (2)	0.017 (7)*	

### (I) 3,5-Dichloro-N-(2,3-dimethylphenyl)benzenesulfonamide Atomic displacement parameters (Å^2^)

**Table:**

	*U*^11^	*U*^22^	*U*^33^	*U*^12^	*U*^13^	*U*^23^
C1	0.0190 (12)	0.0141 (11)	0.0178 (11)	−0.0010 (8)	0.0013 (9)	−0.0029 (9)
C2	0.0169 (12)	0.0171 (12)	0.0225 (12)	−0.0005 (9)	−0.0019 (9)	−0.0001 (9)
C3	0.0289 (14)	0.0171 (12)	0.0202 (13)	0.0006 (10)	−0.0053 (10)	0.0011 (9)
C4	0.0305 (15)	0.0193 (12)	0.0195 (13)	−0.0011 (10)	0.0068 (11)	0.0018 (10)
C5	0.0170 (12)	0.0185 (12)	0.0288 (13)	−0.0018 (9)	0.0041 (10)	−0.0033 (10)
C6	0.0191 (12)	0.0184 (12)	0.0196 (12)	0.0006 (9)	−0.0019 (10)	−0.0019 (9)
C7	0.0242 (13)	0.0164 (12)	0.0194 (12)	−0.0042 (9)	−0.0008 (10)	0.0030 (9)
C8	0.0254 (13)	0.0159 (12)	0.0229 (13)	0.0000 (10)	0.0012 (10)	0.0023 (9)
C9	0.0360 (15)	0.0178 (13)	0.0224 (14)	−0.0012 (10)	−0.0033 (11)	0.0012 (9)
C10	0.0322 (15)	0.0249 (14)	0.0322 (15)	−0.0020 (11)	−0.0100 (12)	0.0009 (11)
C11	0.0212 (13)	0.0273 (14)	0.0395 (16)	−0.0026 (10)	−0.0032 (12)	−0.0011 (12)
C12	0.0243 (13)	0.0208 (13)	0.0296 (14)	−0.0044 (10)	0.0044 (10)	−0.0020 (10)
C13	0.0300 (15)	0.0337 (15)	0.0235 (14)	0.0046 (11)	0.0012 (11)	−0.0033 (11)
C14	0.0444 (18)	0.0376 (17)	0.0225 (14)	0.0008 (13)	−0.0048 (13)	−0.0040 (12)
N1	0.0201 (11)	0.0197 (11)	0.0183 (10)	−0.0014 (8)	0.0001 (8)	0.0010 (8)
O1	0.0220 (9)	0.0251 (10)	0.0243 (9)	−0.0009 (7)	0.0067 (7)	−0.0038 (7)
O2	0.0294 (10)	0.0275 (10)	0.0153 (9)	−0.0031 (7)	−0.0014 (7)	−0.0009 (7)
S1	0.0193 (4)	0.0196 (4)	0.0144 (4)	−0.0022 (2)	0.0020 (3)	−0.0011 (2)
CL1	0.0443 (5)	0.0343 (5)	0.0270 (5)	−0.0034 (3)	−0.0159 (3)	0.0102 (3)
CL2	0.0172 (4)	0.0395 (5)	0.0504 (5)	−0.0054 (2)	0.0073 (3)	0.0049 (3)

### (I) 3,5-Dichloro-N-(2,3-dimethylphenyl)benzenesulfonamide Geometric parameters (Å, º)

**Table:**

C1—C2	1.385 (3)	C9—C10	1.382 (4)
C1—C6	1.392 (3)	C9—C14	1.512 (4)
C1—S1	1.776 (2)	C10—C11	1.384 (4)
C2—C3	1.379 (4)	C10—H10	0.9500
C2—H2	0.9500	C11—C12	1.380 (4)
C3—C4	1.379 (4)	C11—H11	0.9500
C3—CL1	1.741 (3)	C12—H12	0.9500
C4—C5	1.387 (4)	C13—H13A	0.9800
C4—H4	0.9500	C13—H13B	0.9800
C5—C6	1.383 (3)	C13—H13C	0.9800
C5—CL2	1.734 (2)	C14—H14A	0.9800
C6—H6	0.9500	C14—H14B	0.9800
C7—C12	1.390 (4)	C14—H14C	0.9800
C7—C8	1.398 (4)	N1—S1	1.630 (2)
C7—N1	1.446 (3)	N1—H1	0.862 (17)
C8—C9	1.410 (4)	O1—S1	1.4285 (18)
C8—C13	1.501 (4)	O2—S1	1.4346 (19)
			
C2—C1—C6	122.4 (2)	C11—C10—H10	119.3
C2—C1—S1	117.50 (17)	C12—C11—C10	119.8 (2)
C6—C1—S1	120.02 (18)	C12—C11—H11	120.1
C3—C2—C1	117.6 (2)	C10—C11—H11	120.1
C3—C2—H2	121.2	C11—C12—C7	119.4 (2)
C1—C2—H2	121.2	C11—C12—H12	120.3
C2—C3—C4	122.5 (2)	C7—C12—H12	120.3
C2—C3—CL1	118.3 (2)	C8—C13—H13A	109.5
C4—C3—CL1	119.19 (19)	C8—C13—H13B	109.5
C3—C4—C5	117.8 (2)	H13A—C13—H13B	109.5
C3—C4—H4	121.1	C8—C13—H13C	109.5
C5—C4—H4	121.1	H13A—C13—H13C	109.5
C6—C5—C4	122.4 (2)	H13B—C13—H13C	109.5
C6—C5—CL2	119.05 (19)	C9—C14—H14A	109.5
C4—C5—CL2	118.49 (19)	C9—C14—H14B	109.5
C5—C6—C1	117.1 (2)	H14A—C14—H14B	109.5
C5—C6—H6	121.4	C9—C14—H14C	109.5
C1—C6—H6	121.4	H14A—C14—H14C	109.5
C12—C7—C8	121.8 (2)	H14B—C14—H14C	109.5
C12—C7—N1	117.5 (2)	C7—N1—S1	119.12 (16)
C8—C7—N1	120.7 (2)	C7—N1—H1	113.9 (19)
C7—C8—C9	117.8 (2)	S1—N1—H1	111.8 (19)
C7—C8—C13	122.1 (2)	O1—S1—O2	119.99 (11)
C9—C8—C13	120.1 (2)	O1—S1—N1	108.44 (11)
C10—C9—C8	119.8 (2)	O2—S1—N1	106.30 (11)
C10—C9—C14	119.9 (3)	O1—S1—C1	106.41 (11)
C8—C9—C14	120.3 (2)	O2—S1—C1	108.61 (11)
C9—C10—C11	121.4 (3)	N1—S1—C1	106.38 (10)
C9—C10—H10	119.3		
			
C6—C1—C2—C3	0.2 (3)	C13—C8—C9—C14	0.9 (4)
S1—C1—C2—C3	177.68 (17)	C8—C9—C10—C11	0.5 (4)
C1—C2—C3—C4	1.5 (4)	C14—C9—C10—C11	179.3 (3)
C1—C2—C3—CL1	−179.10 (18)	C9—C10—C11—C12	−0.5 (4)
C2—C3—C4—C5	−1.3 (4)	C10—C11—C12—C7	0.0 (4)
CL1—C3—C4—C5	179.28 (19)	C8—C7—C12—C11	0.5 (4)
C3—C4—C5—C6	−0.6 (4)	N1—C7—C12—C11	−179.2 (2)
C3—C4—C5—CL2	178.04 (19)	C12—C7—N1—S1	76.0 (3)
C4—C5—C6—C1	2.2 (4)	C8—C7—N1—S1	−103.7 (2)
CL2—C5—C6—C1	−176.42 (17)	C7—N1—S1—O1	−53.25 (19)
C2—C1—C6—C5	−2.0 (3)	C7—N1—S1—O2	176.48 (17)
S1—C1—C6—C5	−179.40 (17)	C7—N1—S1—C1	60.9 (2)
C12—C7—C8—C9	−0.4 (4)	C2—C1—S1—O1	36.2 (2)
N1—C7—C8—C9	179.3 (2)	C6—C1—S1—O1	−146.31 (18)
C12—C7—C8—C13	179.9 (2)	C2—C1—S1—O2	166.63 (17)
N1—C7—C8—C13	−0.4 (4)	C6—C1—S1—O2	−15.9 (2)
C7—C8—C9—C10	−0.1 (4)	C2—C1—S1—N1	−79.3 (2)
C13—C8—C9—C10	179.6 (2)	C6—C1—S1—N1	98.2 (2)
C7—C8—C9—C14	−178.8 (2)		

### (I) 3,5-Dichloro-N-(2,3-dimethylphenyl)benzenesulfonamide Hydrogen-bond geometry (Å, º)

**Table:**

*D*—H···*A*	*D*—H	H···*A*	*D*···*A*	*D*—H···*A*
N1—H1···O2^i^	0.86	2.14	2.9590	159
C4—H4···O1^ii^	0.95	2.41	3.332 (3)	164

Symmetry codes: (i) −*x*+1, −*y*+1, −*z*+1; (ii) *x*−1/2, −*y*+1/2, *z*+1/2.

### (II) 3,5-Dichloro-N-(2,6-dimethylphenyl)benzenesulfonamide Crystal data

**Table:**

C_14_H_13_Cl_2_NO_2_S	*F*(000) = 340
*M**_r_* = 330.21	Prism
Triclinic, *P*1	*D*_x_ = 1.468 Mg m^−^^3^
Hall symbol: -P 1	Melting point: 445 K
*a* = 8.4817 (15) Å	Cu *K*α radiation, λ = 1.54178 Å
*b* = 8.6149 (15) Å	Cell parameters from 127 reflections
*c* = 12.167 (2) Å	θ = 7.7–63.7°
α = 109.875 (5)°	µ = 5.22 mm^−^^1^
β = 91.900 (5)°	*T* = 100 K
γ = 114.190 (5)°	Prism, colourless
*V* = 747.1 (2) Å^3^	0.29 × 0.26 × 0.22 mm
*Z* = 2	

### (II) 3,5-Dichloro-N-(2,6-dimethylphenyl)benzenesulfonamide Data collection

**Table:**

Bruker APEXII CCD area detector diffractometer	2400 independent reflections
Radiation source: fine-focus sealed tube	1960 reflections with *I* > 2σ(*I*)
Graphite monochromator	*R*_int_ = 0.124
phi and φ scans	θ_max_ = 63.7°, θ_min_ = 7.7°
Absorption correction: multi-scan (SADABS; Bruker, 2009)	*h* = −9→9
*T*_min_ = 0.275, *T*_max_ = 0.317	*k* = −9→9
6977 measured reflections	*l* = −14→14

### (II) 3,5-Dichloro-N-(2,6-dimethylphenyl)benzenesulfonamide Refinement

**Table:**

Refinement on *F*^2^	1 restraint
Least-squares matrix: full	Hydrogen site location: mixed
*R*[*F*^2^ > 2σ(*F*^2^)] = 0.074	H atoms treated by a mixture of independent and constrained refinement
*wR*(*F*^2^) = 0.233	*w* = 1/[σ^2^(*F*_o_^2^) + (0.1757*P*)^2^ + 0.6254*P*] where *P* = (*F*_o_^2^ + 2*F*_c_^2^)/3
*S* = 1.02	(Δ/σ)_max_ < 0.001
2400 reflections	Δρ_max_ = 0.99 e Å^−^^3^
187 parameters	Δρ_min_ = −0.60 e Å^−^^3^

### (II) 3,5-Dichloro-N-(2,6-dimethylphenyl)benzenesulfonamide Special details

**Table:**

Geometry. All esds (except the esd in the dihedral angle between two l.s. planes) are estimated using the full covariance matrix. The cell esds are taken into account individually in the estimation of esds in distances, angles and torsion angles; correlations between esds in cell parameters are only used when they are defined by crystal symmetry. An approximate (isotropic) treatment of cell esds is used for estimating esds involving l.s. planes.

### (II) 3,5-Dichloro-N-(2,6-dimethylphenyl)benzenesulfonamide Fractional atomic coordinates and isotropic or equivalent isotropic displacement parameters (Å^2^)

**Table:**

	*x*	*y*	*z*	*U*_iso_*/*U*_eq_	
C1	0.8417 (4)	0.5358 (5)	0.1002 (4)	0.0203 (9)	
C2	0.7459 (5)	0.3554 (5)	0.0217 (4)	0.0241 (10)	
H2	0.644651	0.320522	−0.034335	0.029*	
C3	0.8001 (5)	0.2253 (6)	0.0260 (4)	0.0249 (10)	
C4	0.9467 (5)	0.2750 (6)	0.1103 (4)	0.0293 (11)	
H4	0.981555	0.185462	0.115199	0.035*	
C5	1.0386 (5)	0.4578 (6)	0.1860 (4)	0.0244 (9)	
C6	0.9904 (5)	0.5912 (6)	0.1831 (4)	0.0238 (10)	
H6	1.056523	0.716677	0.235918	0.029*	
C7	0.6763 (5)	0.7502 (5)	0.3150 (4)	0.0211 (9)	
C8	0.6651 (5)	0.6237 (6)	0.3668 (4)	0.0236 (9)	
C9	0.7175 (5)	0.6907 (7)	0.4904 (5)	0.0317 (10)	
H9	0.712684	0.607766	0.526909	0.038*	
C10	0.7765 (7)	0.8761 (8)	0.5608 (5)	0.0444 (13)	
H10	0.813297	0.919881	0.644715	0.053*	
C11	0.7815 (7)	0.9971 (7)	0.5082 (5)	0.0438 (13)	
H11	0.821341	1.123758	0.557153	0.053*	
C12	0.7300 (6)	0.9386 (6)	0.3863 (4)	0.0316 (10)	
C13	0.5959 (5)	0.4220 (6)	0.2945 (4)	0.0310 (11)	
H13A	0.693639	0.395208	0.269174	0.046*	
H13B	0.509594	0.386272	0.223963	0.046*	
H13C	0.539097	0.352168	0.342708	0.046*	
C14	0.7304 (8)	1.0734 (7)	0.3333 (6)	0.0472 (14)	
H14A	0.728356	1.180297	0.395426	0.071*	
H14B	0.626024	1.013250	0.269485	0.071*	
H14C	0.837145	1.114337	0.300552	0.071*	
N1	0.6231 (4)	0.6893 (4)	0.1889 (3)	0.0201 (8)	
O1	0.9163 (3)	0.8797 (4)	0.1562 (3)	0.0251 (7)	
O2	0.6731 (3)	0.6436 (4)	−0.0152 (3)	0.0230 (7)	
S1	0.76785 (10)	0.70217 (12)	0.10237 (9)	0.0183 (4)	
CL1	0.68515 (13)	−0.00070 (13)	−0.07437 (12)	0.0365 (4)	
CL2	1.22462 (13)	0.52449 (17)	0.28944 (10)	0.0372 (4)	
H1	0.529 (4)	0.590 (4)	0.154 (4)	0.029 (13)*	

### (II) 3,5-Dichloro-N-(2,6-dimethylphenyl)benzenesulfonamide Atomic displacement parameters (Å^2^)

**Table:**

	*U*^11^	*U*^22^	*U*^33^	*U*^12^	*U*^13^	*U*^23^
C1	0.0210 (17)	0.0232 (19)	0.021 (2)	0.0095 (15)	0.0048 (14)	0.0138 (18)
C2	0.0242 (19)	0.028 (2)	0.026 (3)	0.0125 (17)	0.0086 (16)	0.015 (2)
C3	0.0255 (19)	0.025 (2)	0.030 (3)	0.0108 (16)	0.0127 (17)	0.017 (2)
C4	0.036 (2)	0.037 (2)	0.036 (3)	0.0227 (19)	0.018 (2)	0.028 (2)
C5	0.0274 (19)	0.036 (2)	0.020 (3)	0.0176 (17)	0.0061 (16)	0.017 (2)
C6	0.0248 (19)	0.031 (2)	0.021 (3)	0.0132 (17)	0.0061 (16)	0.0152 (19)
C7	0.0243 (18)	0.023 (2)	0.017 (2)	0.0111 (15)	0.0018 (14)	0.0093 (18)
C8	0.0214 (18)	0.027 (2)	0.027 (3)	0.0112 (16)	0.0060 (15)	0.0163 (19)
C9	0.036 (2)	0.039 (2)	0.028 (3)	0.0152 (19)	0.0064 (17)	0.024 (2)
C10	0.054 (3)	0.047 (3)	0.016 (3)	0.009 (2)	0.002 (2)	0.011 (2)
C11	0.071 (3)	0.025 (2)	0.019 (3)	0.012 (2)	0.004 (2)	0.003 (2)
C12	0.046 (2)	0.026 (2)	0.018 (3)	0.0152 (18)	0.0054 (17)	0.0052 (19)
C13	0.037 (2)	0.037 (2)	0.035 (3)	0.0208 (19)	0.0160 (18)	0.025 (2)
C14	0.084 (4)	0.028 (2)	0.038 (4)	0.032 (2)	0.014 (3)	0.014 (2)
N1	0.0240 (16)	0.0213 (17)	0.014 (2)	0.0091 (14)	0.0001 (13)	0.0080 (15)
O1	0.0289 (14)	0.0217 (14)	0.0246 (19)	0.0094 (12)	0.0039 (11)	0.0114 (13)
O2	0.0277 (13)	0.0266 (15)	0.0197 (19)	0.0136 (11)	0.0048 (11)	0.0128 (13)
S1	0.0223 (6)	0.0178 (6)	0.0160 (7)	0.0090 (4)	0.0011 (4)	0.0080 (5)
CL1	0.0340 (6)	0.0203 (6)	0.0533 (10)	0.0120 (5)	0.0106 (5)	0.0121 (6)
CL2	0.0392 (7)	0.0598 (8)	0.0266 (8)	0.0335 (6)	0.0034 (5)	0.0189 (6)

### (II) 3,5-Dichloro-N-(2,6-dimethylphenyl)benzenesulfonamide Geometric parameters (Å, º)

**Table:**

C1—C2	1.375 (6)	C9—C10	1.386 (8)
C1—C6	1.389 (6)	C9—H9	0.9500
C1—S1	1.777 (4)	C10—C11	1.385 (8)
C2—C3	1.390 (6)	C10—H10	0.9500
C2—H2	0.9500	C11—C12	1.383 (7)
C3—C4	1.401 (6)	C11—H11	0.9500
C3—CL1	1.726 (4)	C12—C14	1.506 (7)
C4—C5	1.377 (6)	C13—H13A	0.9800
C4—H4	0.9500	C13—H13B	0.9800
C5—C6	1.379 (6)	C13—H13C	0.9800
C5—CL2	1.743 (4)	C14—H14A	0.9800
C6—H6	0.9500	C14—H14B	0.9800
C7—C8	1.407 (6)	C14—H14C	0.9800
C7—C12	1.417 (6)	N1—S1	1.638 (3)
C7—N1	1.430 (5)	N1—H1	0.85 (2)
C8—C9	1.395 (6)	O1—S1	1.428 (3)
C8—C13	1.493 (6)	O2—S1	1.431 (3)
			
C2—C1—C6	122.0 (4)	C11—C10—H10	120.2
C2—C1—S1	119.5 (3)	C12—C11—C10	121.8 (5)
C6—C1—S1	118.3 (3)	C12—C11—H11	119.1
C1—C2—C3	118.7 (4)	C10—C11—H11	119.1
C1—C2—H2	120.7	C11—C12—C7	117.9 (5)
C3—C2—H2	120.7	C11—C12—C14	120.1 (4)
C2—C3—C4	120.9 (4)	C7—C12—C14	122.0 (4)
C2—C3—CL1	119.4 (3)	C8—C13—H13A	109.5
C4—C3—CL1	119.6 (3)	C8—C13—H13B	109.5
C5—C4—C3	117.8 (4)	H13A—C13—H13B	109.5
C5—C4—H4	121.1	C8—C13—H13C	109.5
C3—C4—H4	121.1	H13A—C13—H13C	109.5
C6—C5—C4	122.8 (4)	H13B—C13—H13C	109.5
C6—C5—CL2	118.4 (3)	C12—C14—H14A	109.5
C4—C5—CL2	118.8 (3)	C12—C14—H14B	109.5
C5—C6—C1	117.6 (4)	H14A—C14—H14B	109.5
C5—C6—H6	121.2	C12—C14—H14C	109.5
C1—C6—H6	121.2	H14A—C14—H14C	109.5
C8—C7—C12	121.2 (4)	H14B—C14—H14C	109.5
C8—C7—N1	120.7 (4)	C7—N1—S1	120.9 (2)
C12—C7—N1	118.0 (4)	C7—N1—H1	118 (4)
C9—C8—C7	118.2 (4)	S1—N1—H1	109 (3)
C9—C8—C13	119.6 (4)	O1—S1—O2	120.06 (18)
C7—C8—C13	122.2 (4)	O1—S1—N1	108.41 (17)
C10—C9—C8	121.2 (5)	O2—S1—N1	106.27 (16)
C10—C9—H9	119.4	O1—S1—C1	107.28 (17)
C8—C9—H9	119.4	O2—S1—C1	107.33 (18)
C9—C10—C11	119.6 (5)	N1—S1—C1	106.81 (17)
C9—C10—H10	120.2		
			
C6—C1—C2—C3	0.1 (6)	C9—C10—C11—C12	−0.4 (8)
S1—C1—C2—C3	−176.4 (3)	C10—C11—C12—C7	−1.9 (8)
C1—C2—C3—C4	1.6 (6)	C10—C11—C12—C14	177.5 (5)
C1—C2—C3—CL1	−178.3 (3)	C8—C7—C12—C11	3.8 (6)
C2—C3—C4—C5	−2.2 (6)	N1—C7—C12—C11	180.0 (4)
CL1—C3—C4—C5	177.7 (3)	C8—C7—C12—C14	−175.5 (4)
C3—C4—C5—C6	1.1 (6)	N1—C7—C12—C14	0.7 (6)
C3—C4—C5—CL2	−178.5 (3)	C8—C7—N1—S1	−96.3 (4)
C4—C5—C6—C1	0.5 (6)	C12—C7—N1—S1	87.5 (4)
CL2—C5—C6—C1	−179.9 (3)	C7—N1—S1—O1	−45.5 (3)
C2—C1—C6—C5	−1.1 (6)	C7—N1—S1—O2	−175.8 (3)
S1—C1—C6—C5	175.4 (3)	C7—N1—S1—C1	69.8 (3)
C12—C7—C8—C9	−3.4 (5)	C2—C1—S1—O1	−159.3 (3)
N1—C7—C8—C9	−179.5 (3)	C6—C1—S1—O1	24.1 (4)
C12—C7—C8—C13	175.3 (4)	C2—C1—S1—O2	−29.0 (4)
N1—C7—C8—C13	−0.8 (5)	C6—C1—S1—O2	154.4 (3)
C7—C8—C9—C10	1.0 (6)	C2—C1—S1—N1	84.6 (4)
C13—C8—C9—C10	−177.7 (4)	C6—C1—S1—N1	−92.0 (3)
C8—C9—C10—C11	0.9 (7)		

### (II) 3,5-Dichloro-N-(2,6-dimethylphenyl)benzenesulfonamide Hydrogen-bond geometry (Å, º)

Cg1 is the centroid of the C1–C6 ring.

**Table:**

*D*—H···*A*	*D*—H	H···*A*	*D*···*A*	*D*—H···*A*
C14—H14*C*···O1	0.98	2.53	3.139 (8)	120
N1—H1···O2^i^	0.85 (4)	2.12 (4)	2.937 (5)	160 (4)
C13—H13*A*···*Cg*1	0.98	2.67	3.493 (5)	142

Symmetry code: (i) −*x*+1, −*y*+1, −*z*.

### (III) 3,5-dichloro-N-(3,5-dimethylphenyl)benzenesulfonamide Crystal data

**Table:**

C_14_H_13_Cl_2_NO_2_S	Prism
*M**_r_* = 330.21	*D*_x_ = 1.496 Mg m^−^^3^
Monoclinic, *P*2_1_/*c*	Melting point: 462 K
Hall symbol: -P 2ybc	Cu *K*α radiation, λ = 1.54178 Å
*a* = 12.2268 (6) Å	Cell parameters from 128 reflections
*b* = 7.0399 (3) Å	θ = 6.8–64.4°
*c* = 17.3130 (8) Å	µ = 5.32 mm^−^^1^
β = 100.409 (1)°	*T* = 100 K
*V* = 1465.70 (12) Å^3^	Prism, colourless
*Z* = 4	0.27 × 0.24 × 0.21 mm
*F*(000) = 680	

### (III) 3,5-dichloro-N-(3,5-dimethylphenyl)benzenesulfonamide Data collection

**Table:**

Bruker APEXII CCD area detector diffractometer	2412 independent reflections
Radiation source: fine-focus sealed tube	2374 reflections with *I* > 2σ(*I*)
Graphite monochromator	*R*_int_ = 0.056
phi and φ scans	θ_max_ = 64.4°, θ_min_ = 6.8°
Absorption correction: multi-scan (SADABS; Bruker,2009)	*h* = −14→14
*T*_min_ = 0.297, *T*_max_ = 0.327	*k* = −8→7
11468 measured reflections	*l* = −19→20

### (III) 3,5-dichloro-N-(3,5-dimethylphenyl)benzenesulfonamide Refinement

**Table:**

Refinement on *F*^2^	1 restraint
Least-squares matrix: full	Hydrogen site location: mixed
*R*[*F*^2^ > 2σ(*F*^2^)] = 0.058	H atoms treated by a mixture of independent and constrained refinement
*wR*(*F*^2^) = 0.152	*w* = 1/[σ^2^(*F*_o_^2^) + (0.1156*P*)^2^ + 2.094*P*] where *P* = (*F*_o_^2^ + 2*F*_c_^2^)/3
*S* = 0.99	(Δ/σ)_max_ = 0.001
2412 reflections	Δρ_max_ = 0.82 e Å^−^^3^
187 parameters	Δρ_min_ = −0.88 e Å^−^^3^

### (III) 3,5-dichloro-N-(3,5-dimethylphenyl)benzenesulfonamide Special details

**Table:**

Geometry. All esds (except the esd in the dihedral angle between two l.s. planes) are estimated using the full covariance matrix. The cell esds are taken into account individually in the estimation of esds in distances, angles and torsion angles; correlations between esds in cell parameters are only used when they are defined by crystal symmetry. An approximate (isotropic) treatment of cell esds is used for estimating esds involving l.s. planes.

### (III) 3,5-dichloro-N-(3,5-dimethylphenyl)benzenesulfonamide Fractional atomic coordinates and isotropic or equivalent isotropic displacement parameters (Å^2^)

**Table:**

	*x*	*y*	*z*	*U*_iso_*/*U*_eq_	
C1	0.4234 (2)	0.1933 (4)	0.62264 (15)	0.0107 (6)	
C2	0.5280 (2)	0.2511 (4)	0.61112 (15)	0.0120 (6)	
H2	0.586016	0.277777	0.654187	0.014*	
C3	0.5443 (2)	0.2682 (4)	0.53442 (15)	0.0120 (6)	
C4	0.4606 (2)	0.2341 (4)	0.47057 (15)	0.0138 (6)	
H4	0.473647	0.246812	0.418356	0.017*	
C5	0.3574 (2)	0.1810 (4)	0.48539 (16)	0.0133 (6)	
C6	0.3366 (2)	0.1572 (4)	0.56068 (16)	0.0128 (6)	
H6	0.265674	0.117738	0.569706	0.015*	
C7	0.2216 (2)	0.4234 (4)	0.69407 (14)	0.0108 (6)	
C8	0.2040 (2)	0.5888 (4)	0.64942 (15)	0.0126 (6)	
H8	0.264930	0.668527	0.644084	0.015*	
C9	0.0966 (2)	0.6368 (4)	0.61262 (16)	0.0146 (6)	
C10	0.0089 (2)	0.5168 (4)	0.62049 (15)	0.0161 (6)	
H10	−0.063981	0.547186	0.593959	0.019*	
C11	0.0254 (2)	0.3533 (4)	0.66637 (16)	0.0144 (6)	
C12	0.1329 (2)	0.3073 (4)	0.70360 (15)	0.0124 (6)	
H12	0.145662	0.196778	0.735448	0.015*	
C13	0.0756 (3)	0.8176 (4)	0.5651 (2)	0.0242 (7)	
H13A	−0.000712	0.816863	0.535489	0.036*	
H13B	0.127963	0.825736	0.528605	0.036*	
H13C	0.085778	0.927260	0.600562	0.036*	
C14	−0.0718 (2)	0.2318 (5)	0.67730 (18)	0.0244 (7)	
H14A	−0.117650	0.300752	0.708934	0.037*	
H14B	−0.044640	0.113872	0.704178	0.037*	
H14C	−0.116566	0.201609	0.625895	0.037*	
N1	0.33273 (18)	0.3807 (3)	0.73296 (12)	0.0111 (5)	
O1	0.49846 (16)	0.1842 (3)	0.77286 (11)	0.0169 (5)	
O2	0.31742 (16)	0.0286 (3)	0.72152 (11)	0.0159 (5)	
S1	0.39385 (5)	0.18095 (9)	0.71907 (3)	0.0102 (3)	
CL1	0.67420 (5)	0.33657 (9)	0.51672 (4)	0.0189 (3)	
CL2	0.24931 (6)	0.14477 (11)	0.40666 (4)	0.0212 (3)	
H1	0.379 (3)	0.475 (4)	0.739 (2)	0.027 (9)*	

### (III) 3,5-dichloro-N-(3,5-dimethylphenyl)benzenesulfonamide Atomic displacement parameters (Å^2^)

**Table:**

	*U*^11^	*U*^22^	*U*^33^	*U*^12^	*U*^13^	*U*^23^
C1	0.0138 (14)	0.0084 (12)	0.0104 (13)	0.0043 (10)	0.0040 (10)	0.0001 (9)
C2	0.0122 (13)	0.0108 (14)	0.0122 (13)	0.0013 (10)	0.0003 (10)	0.0004 (10)
C3	0.0135 (13)	0.0064 (13)	0.0175 (14)	0.0022 (10)	0.0066 (11)	0.0016 (10)
C4	0.0214 (14)	0.0108 (13)	0.0105 (13)	0.0039 (11)	0.0067 (11)	0.0022 (10)
C5	0.0161 (14)	0.0116 (14)	0.0111 (14)	0.0037 (10)	−0.0010 (11)	−0.0026 (10)
C6	0.0122 (13)	0.0117 (13)	0.0153 (14)	−0.0003 (9)	0.0043 (11)	0.0001 (10)
C7	0.0122 (13)	0.0148 (14)	0.0060 (11)	0.0012 (10)	0.0036 (10)	−0.0044 (10)
C8	0.0132 (13)	0.0133 (13)	0.0131 (13)	−0.0017 (10)	0.0071 (10)	−0.0018 (10)
C9	0.0170 (14)	0.0159 (14)	0.0116 (13)	0.0027 (11)	0.0044 (11)	0.0020 (10)
C10	0.0122 (13)	0.0231 (15)	0.0124 (13)	0.0024 (11)	0.0004 (10)	0.0007 (11)
C11	0.0137 (14)	0.0188 (14)	0.0109 (13)	−0.0026 (11)	0.0027 (11)	−0.0014 (10)
C12	0.0142 (13)	0.0157 (14)	0.0072 (12)	0.0007 (10)	0.0019 (10)	0.0006 (10)
C13	0.0195 (15)	0.0220 (16)	0.0321 (18)	0.0041 (12)	0.0072 (13)	0.0111 (13)
C14	0.0128 (13)	0.0310 (17)	0.0271 (16)	−0.0065 (13)	−0.0021 (12)	0.0065 (13)
N1	0.0103 (11)	0.0123 (12)	0.0108 (11)	−0.0017 (9)	0.0021 (9)	−0.0031 (9)
O1	0.0134 (10)	0.0264 (11)	0.0097 (10)	0.0052 (8)	−0.0014 (8)	0.0020 (8)
O2	0.0191 (10)	0.0123 (10)	0.0179 (10)	−0.0004 (8)	0.0072 (8)	0.0037 (7)
S1	0.0103 (4)	0.0134 (4)	0.0068 (4)	0.0015 (2)	0.0015 (3)	0.0016 (2)
CL1	0.0142 (4)	0.0199 (4)	0.0255 (5)	−0.0004 (2)	0.0112 (3)	0.0027 (2)
CL2	0.0202 (4)	0.0311 (5)	0.0103 (4)	−0.0013 (3)	−0.0029 (3)	−0.0028 (3)

### (III) 3,5-dichloro-N-(3,5-dimethylphenyl)benzenesulfonamide Geometric parameters (Å, º)

**Table:**

C1—C6	1.389 (4)	C9—C10	1.391 (4)
C1—C2	1.391 (4)	C9—C13	1.512 (4)
C1—S1	1.773 (3)	C10—C11	1.393 (4)
C2—C3	1.383 (4)	C10—H10	0.9500
C2—H2	0.9500	C11—C12	1.394 (4)
C3—C4	1.385 (4)	C11—C14	1.504 (4)
C3—CL1	1.739 (3)	C12—H12	0.9500
C4—C5	1.385 (4)	C13—H13A	0.9800
C4—H4	0.9500	C13—H13B	0.9800
C5—C6	1.382 (4)	C13—H13C	0.9800
C5—CL2	1.737 (3)	C14—H14A	0.9800
C6—H6	0.9500	C14—H14B	0.9800
C7—C12	1.392 (4)	C14—H14C	0.9800
C7—C8	1.393 (4)	N1—S1	1.631 (2)
C7—N1	1.435 (3)	N1—H1	0.869 (19)
C8—C9	1.394 (4)	O1—S1	1.440 (2)
C8—H8	0.9500	O2—S1	1.428 (2)
			
C6—C1—C2	122.5 (2)	C11—C10—H10	119.2
C6—C1—S1	117.4 (2)	C10—C11—C12	118.9 (3)
C2—C1—S1	119.9 (2)	C10—C11—C14	120.3 (3)
C3—C2—C1	117.3 (2)	C12—C11—C14	120.7 (3)
C3—C2—H2	121.4	C7—C12—C11	119.9 (2)
C1—C2—H2	121.4	C7—C12—H12	120.0
C4—C3—C2	122.6 (2)	C11—C12—H12	120.0
C4—C3—CL1	118.27 (19)	C9—C13—H13A	109.5
C2—C3—CL1	119.2 (2)	C9—C13—H13B	109.5
C3—C4—C5	117.7 (2)	H13A—C13—H13B	109.5
C3—C4—H4	121.1	C9—C13—H13C	109.5
C5—C4—H4	121.1	H13A—C13—H13C	109.5
C6—C5—C4	122.4 (3)	H13B—C13—H13C	109.5
C6—C5—CL2	118.6 (2)	C11—C14—H14A	109.5
C4—C5—CL2	118.9 (2)	C11—C14—H14B	109.5
C5—C6—C1	117.5 (3)	H14A—C14—H14B	109.5
C5—C6—H6	121.3	C11—C14—H14C	109.5
C1—C6—H6	121.3	H14A—C14—H14C	109.5
C12—C7—C8	120.8 (2)	H14B—C14—H14C	109.5
C12—C7—N1	120.9 (2)	C7—N1—S1	122.12 (18)
C8—C7—N1	118.3 (2)	C7—N1—H1	116 (2)
C7—C8—C9	119.7 (2)	S1—N1—H1	112 (2)
C7—C8—H8	120.2	O2—S1—O1	120.14 (12)
C9—C8—H8	120.2	O2—S1—N1	108.89 (11)
C10—C9—C8	119.2 (2)	O1—S1—N1	105.56 (11)
C10—C9—C13	120.4 (3)	O2—S1—C1	108.09 (12)
C8—C9—C13	120.4 (3)	O1—S1—C1	107.45 (12)
C9—C10—C11	121.5 (2)	N1—S1—C1	105.85 (11)
C9—C10—H10	119.2		
			
C6—C1—C2—C3	−1.3 (4)	C9—C10—C11—C12	−1.5 (4)
S1—C1—C2—C3	−176.48 (19)	C9—C10—C11—C14	176.7 (3)
C1—C2—C3—C4	1.4 (4)	C8—C7—C12—C11	1.9 (4)
C1—C2—C3—CL1	−179.05 (19)	N1—C7—C12—C11	178.9 (2)
C2—C3—C4—C5	0.0 (4)	C10—C11—C12—C7	−0.5 (4)
CL1—C3—C4—C5	−179.60 (19)	C14—C11—C12—C7	−178.7 (2)
C3—C4—C5—C6	−1.5 (4)	C12—C7—N1—S1	59.0 (3)
C3—C4—C5—CL2	177.90 (19)	C8—C7—N1—S1	−123.9 (2)
C4—C5—C6—C1	1.5 (4)	C7—N1—S1—O2	−44.7 (2)
CL2—C5—C6—C1	−177.86 (19)	C7—N1—S1—O1	−174.93 (19)
C2—C1—C6—C5	−0.1 (4)	C7—N1—S1—C1	71.3 (2)
S1—C1—C6—C5	175.20 (19)	C6—C1—S1—O2	37.4 (2)
C12—C7—C8—C9	−1.2 (4)	C2—C1—S1—O2	−147.2 (2)
N1—C7—C8—C9	−178.3 (2)	C6—C1—S1—O1	168.42 (19)
C7—C8—C9—C10	−0.7 (4)	C2—C1—S1—O1	−16.2 (2)
C7—C8—C9—C13	178.9 (2)	C6—C1—S1—N1	−79.1 (2)
C8—C9—C10—C11	2.1 (4)	C2—C1—S1—N1	96.2 (2)
C13—C9—C10—C11	−177.5 (3)		

### (III) 3,5-dichloro-N-(3,5-dimethylphenyl)benzenesulfonamide Hydrogen-bond geometry (Å, º)

Cg2 is the centroid of the aniline ring C7–C12

**Table:**

*D*—H···*A*	*D*—H	H···*A*	*D*···*A*	*D*—H···*A*
N1—H1···O1^i^	0.87	2.13	2.9848	167
C14—H14*B*···*Cg*2^ii^	0.98	2.86	3.5135	124

Symmetry codes: (i) −*x*+1, *y*+1/2, −*z*+3/2; (ii) −*x*, *y*+1/2, −*z*+1/2.
